# Green Tea Polyphenols Upregulate the Nrf2 Signaling Pathway and Suppress Oxidative Stress and Inflammation Markers in D-Galactose-Induced Liver Aging in Mice

**DOI:** 10.3389/fnut.2022.836112

**Published:** 2022-02-23

**Authors:** Dongxu Wang, Taotao Wang, Zhanming Li, Yuanxin Guo, Daniel Granato

**Affiliations:** ^1^School of Grain Science and Technology, Jiangsu University of Science and Technology, Zhenjiang, China; ^2^Department of Clinical Nutrition, Affiliated Hospital of Jiangsu University, Zhenjiang, China; ^3^Department of Biological Sciences, Faculty of Science and Engineering, University of Limerick, Limerick, Ireland

**Keywords:** green tea polyphenols (GTPs), aging, antioxidants, D-galactose, inflammatory cytokines, Nrf2 signaling pathway

## Abstract

The beneficial effects of green tea polyphenols (GTPs) on D-galactose (D-Gal)-induced liver aging in male Kunming mice were investigated. For this purpose, 40 adult male Kunming mice were divided into four groups. All animals, except for the normal control and GTPs control, were intraperitoneally injected with D-galactose (D-Gal; 300 mg/kg/day for 5 days a week) for 12 consecutive weeks, and the D-Gal-treated mice were allowed free access to 0.05% GTPs (w/w) diet or normal diet for 12 consecutive weeks. Results showed that GTP administration improved the liver index and decreased transaminases and total bilirubin levels. Furthermore, GTPs significantly increased hepatic glutathione and total antioxidant levels, and the activities of superoxide dismutase, catalase, and glutathione *S*-transferase (GST). Furthermore, GTPs downregulated 8-hydroxy-2-deoxyguanosine, advanced glycation end products, and hepatic oxidative stress markers, such as malondialdehyde and nitric oxide. Additionally, GTPs abrogated dysregulation in hepatic Kelch-like ECH-associated protein 1 and nuclear factor erythroid 2-related factor 2 (Nrf2) and its downstream target gene expression [heme oxygenase 1, NAD(P)H:quinone oxidoreductase 1, and GST] and inhibited tumor necrosis factor-α, transforming growth factor-β, and interleukin (IL)-1β and IL-6 in the liver of treated mice. Finally, GTPs effectively attenuated D-Gal-induced edema, vacuole formation, and inflammatory cell infiltration. In conclusion, GTPs showed antioxidant and anti-inflammatory properties in D-Gal-induced aging mice, and may be considered a natural alternative to the effects of hepatic aging.

## Introduction

Aging is one kind of irreversible and perennial natural biological process that accounts for genetic, internal, and external environmental factors. This process is characterized by a progressive loss of physiological integrity, which invariably leads to impairments in the organizational structure and function of organs (1). The aging of tissues/organs makes the human body susceptible to adverse circumstances and is the primary risk factor for major human pathologies (2). Like other organs, after the growth and development, the liver undergoes a series of degeneration processes, such as aging, that encompasses changes in its morphological structure to metabolic functions (3). Aging-related liver diseases mainly include alterations of hepatic structure and function, where the increase of liver volume and decrease of hepatic blood flow and perfusion occur. These changes increase the liver fibrosis, hepatocarcinoma, and mortality rate of susceptible elderly people and can thus be considered adverse prognostic factors (3–5). At the cellular level, the disturbances of proteostasis by protein oxidation aggregates trigger reactive oxygen species (ROS) and inflammatory cytokines production (6, 7). Liver aging is usually manifested as a decrease in albumin (Alb) levels and an increase in total bilirubin (TBiL), alkaline phosphatase (ALP), and aminotransferase levels in the blood (8). In addition, liver aging may be related to cytoplasmic polyploidy and decreases in the surface area of the endoplasmic reticulum and the number of mitochondria, thus imposing a negative impact on the functions of hepatocytes (4). Similarly, the damage of hepatocyte mitochondrial function increases the incidence of autoimmune and other age-related diseases (9). Thus, strategies to counteract/alleviate the harmful effects of aging on liver function are desired from the public health perspective.

Green tea has been one of the most consumed non-alcoholic beverages in more than 160 countries (10). The beneficial health effects of green tea are generally associated with its polyphenols, which may account for up to 30% of its dry weight (10). Green tea polyphenols (GTPs) are mainly composed of monomeric flavan-3-ols, such as catechins (10). GTPs exhibit numerous biological effects; for example, they have anti-obesity, anti-inflammatory, antioxidant, neuroprotective, and antitumor properties (10–12). Most biological effects of GTPs are attributed to their ability to transcriptionally upregulate the nuclear factor erythroid 2-related factor 2 (Nrf2)/Kelch-like ECH-associated protein 1 (Keap1) signaling pathway in response to the regulation of antioxidant and Phase II detoxification enzymes and nuclear factor-kappa β (NF-κβ) in different organs, particularly in liver tissue (13, 14).

To our knowledge, the protective effects of GTPs on liver aging have not been extensively evaluated and findings obtained with different *in vivo* protocols are inconclusive and not convergent. For instance, a previous study has shown that in an adult male Sprague–Dawley rat model of metabolic syndrome induced by a high-fat diet, GTPs were able to decrease liver transaminases, oxidative markers, and inflammatory cytokines in the liver (15). Conversely, in a C57BL/6 mice model of cholesterol-induced steatohepatitis, Hirsch et al. observed that GTPs exacerbated hepatic steatosis, oxidative stress, bile acids, and liver damage (16). Thus, it is of pivotal importance to understand the mechanisms of how GTPs can affect inflammation and oxidative stress using different protocols. Considering the global trend for natural products that can be used as adjuvant agents to decrease the risk of diseases and the scientific gap on the beneficial effects of GTPs on liver aging, this work focused on the effects of GTPs on D-galactose (D-Gal)-induced aging in male Kunming mouse liver. The underpinning mechanisms of action were unveiled by quantifying the main oxidative, inflammation, and senescence markers.

## Materials and Methods

### Chemicals and Reagents

D-Gal (CAS: 59-23-4) of 99% purity was purchased from Sigma–Aldrich Chemical Co. (MO, United States). GTPs [gallic acid (4.9%), catechin (0.42%), epicatechin (4.17%), gallocatechin gallate (1.28%), epigallocatechin (10.76%), epicatechin gallate (7.46%), epigallocatechin-3-gallate (60.97%), anthocyanins (3.42%), leukoanthocyanins (1.34%), and other phenolic acids (5.28%)] were purchased from Hefei Jishi Mingxiang Biotechnology Co. Ltd. (Anhui, China). Commercial kits for measuring the levels of alanine aminotransferase (ALT), aspartate aminotransferase (AST), ALP, Alb, TBiL, total superoxide dismutase (T-SOD), catalase (CAT), glutathione *S*-transferase (GST), total antioxidant capacity (T-AOC), glutathione (GSH), malondialdehyde (MDA), nitric oxide (NO), and ELISA kits for measuring the levels of 8-hydroxy-2-deoxyguanosine (8-OHdG) and advanced glycation end products (AGEs) were obtained from Nanjing Jiancheng Bioengineering Institute (Nanjing, China). Mouse interleukin (IL)-1β, IL-6, tumor necrosis factor (TNF)-α, transforming growth factor (TGF)-β, and heme oxygenase 1 (HO-1) ELISA kits were purchased from Sigma–Aldrich (MO, United States), Invitrogen (CA, United States), BD Biosciences (CA, United States), Cell Sciences Inc. (MA, United States), and Abcam (CA, United States), respectively. Mouse Nrf2, Kelch-like ECH-associated protein 1 (Keap1), and NAD(P)H:quinone oxidoreductase 1 (NQO1) ELISA kits were purchased from CUSABIO (Wuhan, China).

### Animals

Healthy male Kunming mice (age: 7–8 weeks, body weight: ~18–22 g) were obtained from Shanghai SLAC Laboratory Animal Co. Ltd. Mice were given a standard laboratory diet and water *ad libitum*.

### Experimental Design for the *in vivo* Protocol

After a week of acclimation, 40 mice (10 in each group) were randomly divided into the following groups: (i) normal control, (ii) D-Gal model, (iii) GTPs intervention, and (iv) GTPs control. The normal control group had free access to the standard diet and received intraperitoneal injections of normal saline for 12 weeks. The D-Gal model group mice that were fed the standard diet had intraperitoneal injections of 300 mg/kg D-Gal (5 days a week) for 12 weeks, the GTPs intervention group mice received a 0.05% GTPs (w/w) diet with the intraperitoneal injection of 300 mg/kg D-Gal for 12 weeks, and the GTPs control group received a 0.05% GTPs (w/w) diet with an intraperitoneal injection of normal saline (5 days a week) for 12 weeks. The mice were anesthetized and sacrificed as per ethical guidelines 24 h after the last administration. The liver of each animal was removed, weighed carefully, and quickly placed into ice-cold phosphate-buffered saline (PBS; 150 mM, pH 7.2). The right liver lobes were fixed with 10% neutral-buffered formalin for >72 h before the preparation of tissue sections for histological examination; the left liver lobes were removed and then stored at −80°C until biochemical analysis. Blood samples were obtained to determine cytokines levels by using ELISA and to evaluate plasma enzyme activities.

### Assessment of Hepatic Function

The plasma levels of hematological biomarkers, including Alb, TBiL, ALP, ALT, and AST, were estimated using an enzyme-labeled instrument and the commercially available colorimetric assay kits according to the manufacturer's protocols. The liver index was calculated according to the following equation: liver weight (g)/body weight (g) × 100%.

### Assessment of Hepatic Antioxidant Markers

The levels or activities of GSH, T-AOC, T-SOD, CAT, and GST in the liver homogenates were measured using an enzyme-labeled instrument by the commercially available colorimetric assay kits according to the manufacturer's protocols.

### Assessment of Hepatic Oxidative Stress and Senescence Markers

The levels of MDA, NO, 8-OhdG, and AGEs in liver homogenates were detected using an enzyme-labeled instrument by the commercially available colorimetric assay kits following the manufacturer's protocol.

### Assessment of Hepatic Inflammatory Mediator Concentrations

The hepatic inflammatory cytokines concentrations, including IL-1β, IL-6, TNF-α, and TGF-β, were assessed using an enzyme-labeled instrument by the commercially available ELISA kits according to the manufacturer's instructions.

### Histopathological Examinations

Fixed liver tissues were embedded in paraffin and cut into coronal sections (4 μm thick), which were then stained with hematoxylin and eosin (H&E) according to Wang et al. (17).

### RNA Isolation and qPCR

The total RNA isolation, reverse transcription, and quantitative real-time reverse transcription–polymerase chain reaction (qRT-PCR) were performed according to the standard protocol described by Wang et al. (17). The sequences of HO-1, NQO1, GST m1, and GST a1 were designed and synthesized as described by Wang et al. (17).

### Preparation of Cytosolic and Nuclear Fractions and Assessment of the Hepatic Nrf2 Pathway

Hepatic cytosolic and nuclear fractions were prepared using a commercially available Minute Cytosolic and Nuclear Extraction Kit (Invent Biotechnologies, Inc.) according to the manufacturer's protocol. Briefly, liver tissues (25 mg/mouse) were washed once with cold buffer solution A on ice for 5 min, and the samples were twisted and ground with a grinding pestle for 1 min (40–60 times). Homogenates were centrifuged at 14,000 g for 5 min, the supernatants were recovered as cytosolic fractions, and the nuclear pellets were lysed in cold buffer solution B on ice for 5 min. After centrifugation at 500 g for 5 min, the nuclei were resuspended in cold buffer solution N for 5 min. Thereafter, 50 mg of protein extract powder and cold buffer solution A were added, and the solution was mixed for 1 min. Hepatic homogenates were centrifuged at 10,000 g for 5 min, and the supernatants were recovered as nuclear fractions for a subsequent ELISA.

### Statistical Analysis

Data are presented as mean ± standard error of the mean. One-way analysis of variance (ANOVA) followed by Tukey's multiple comparison *post-hoc* test were used to compare the treatments. *P* < 0.05 were considered statistically significant.

## Results

### Effects of GTPs on the Liver Function Markers

The beneficial effects of GTPs on liver function were investigated by assessing organ index and multiple hematological indicators. The results in Table 1 and Figure 1 show that the liver index and hematological indicators were significantly increased after the exposure to D-Gal, whereas the liver index and the hematological indicators were significantly lower in the GTP-treated group. In particular, plasmatic ALP, ALT, and AST levels of the D-Gal mice were significantly increased (Figure 1C), revealing that the liver function in the D-Gal model group was disrupted. Moreover, plasmatic ALP, ALT, and AST levels were significantly lower in the D-Gal-treated mice supplemented with GTPs. In addition, plasmatic Alb level was 24% lower and plasma TBiL level was 92% higher in the D-Gal model group compared with the normal control group. Furthermore, plasmatic Alb and plasma TBiL levels were significantly higher and lower, respectively, in the D-Gal model mouse were supplemented with GTPs (Figures 1A,B). However, the administration of GTPs did not affect the liver index and liver damage indexes.

**Table 1:** Effects of GTPs on body weight and liver indexes.

**Groups**	**Body weight**		**Liver weight (g)**	**Liver index (%)**
	**Initial**	**Final**		
Normal control	24.8 ± 1.8	37.2 ± 2.6	1.92 ± 0.14	5.16 ± 0.28
D-Gal model	25.2 ± 1.8	36.5 ± 2.2	2.45 ± 0.16^**^	6.71 ± 0.35^*^
GTPs intervention	25.0 ± 1.6	37.8 ± 2.4	2.23 ± 0.12	5.90 ± 0.31^#^
GTPs control	25.1 ± 1.7	37.9 ± 2.5	2.02 ± 0.13	5.32 ± 0.26

*D-Gal, D-galactose; GTPs, green tea polyphenols*.

* *P < 0.05 and*

** *P < 0.01 vs. normal control group*;

# *P < 0.05 vs. D-Gal model group*.

**Figure 1:**
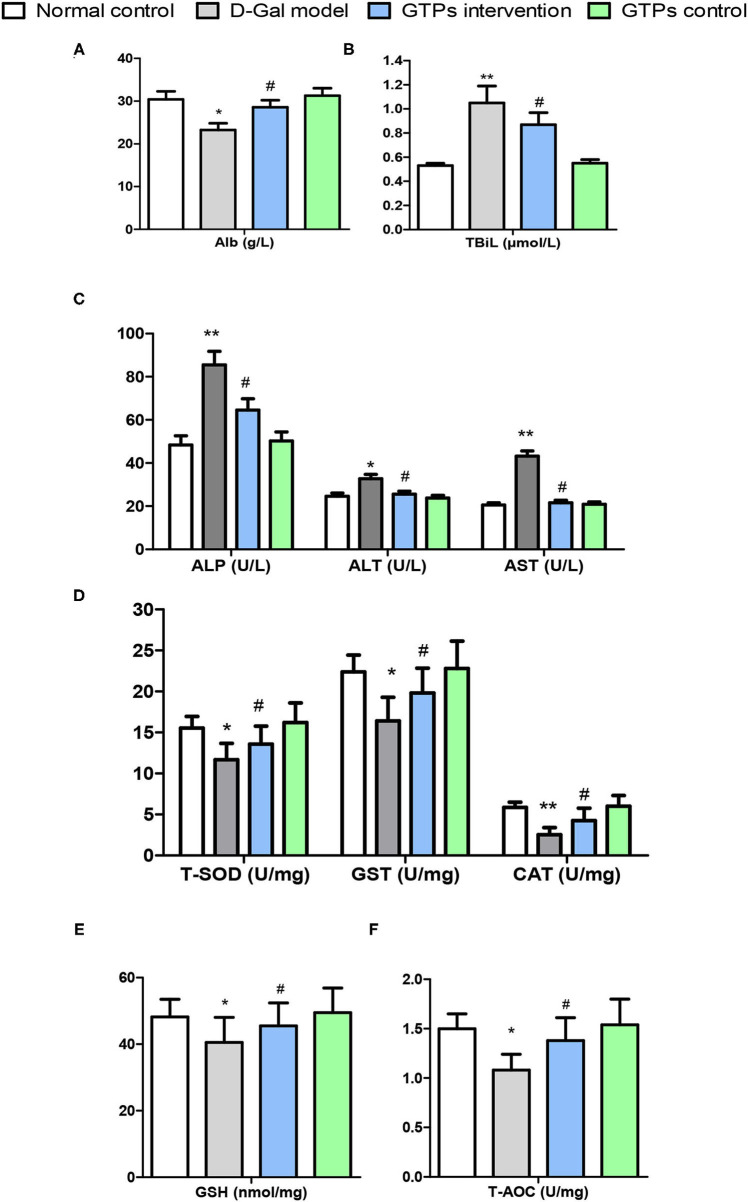
Effects of GTPs on liver damage indexes and antioxidant markers of D-gal treated mice. **(A)** Plasma levels of Alb. **(B)** Plasma levels of TBiL. **(C)** Plasma levels of ALP, ALT, and AST. **(D)** Hepatic contents of T-SOD, GST, and CAT. **(E)** Hepatic levels of GSH. **(F)** Hepatic levels of T-AOC. ^*^P < 0.05 and ^**^P < 0.001 vs. control group; ^#^*P* < 0.05 and ^##^*P* < 0.01 vs. D-Gal model group. Alb, albumin; ALP, alkaline phosphatase; ALT, alanine aminotransferase; AST, aspartate aminotransferase; CAT, catalase; D-Gal, D-galactose; GSH, reduced glutathione; GST, glutathione S-transferase; GTPs, green tea polyphenols; T-SOD, total superoxide dismutase; T-AOC, total antioxidant capacity; TBil, total bilirubin.

### Effects of GTPs on Liver Antioxidant, Oxidative Stress, and Senescence Markers

**Figure 2:**
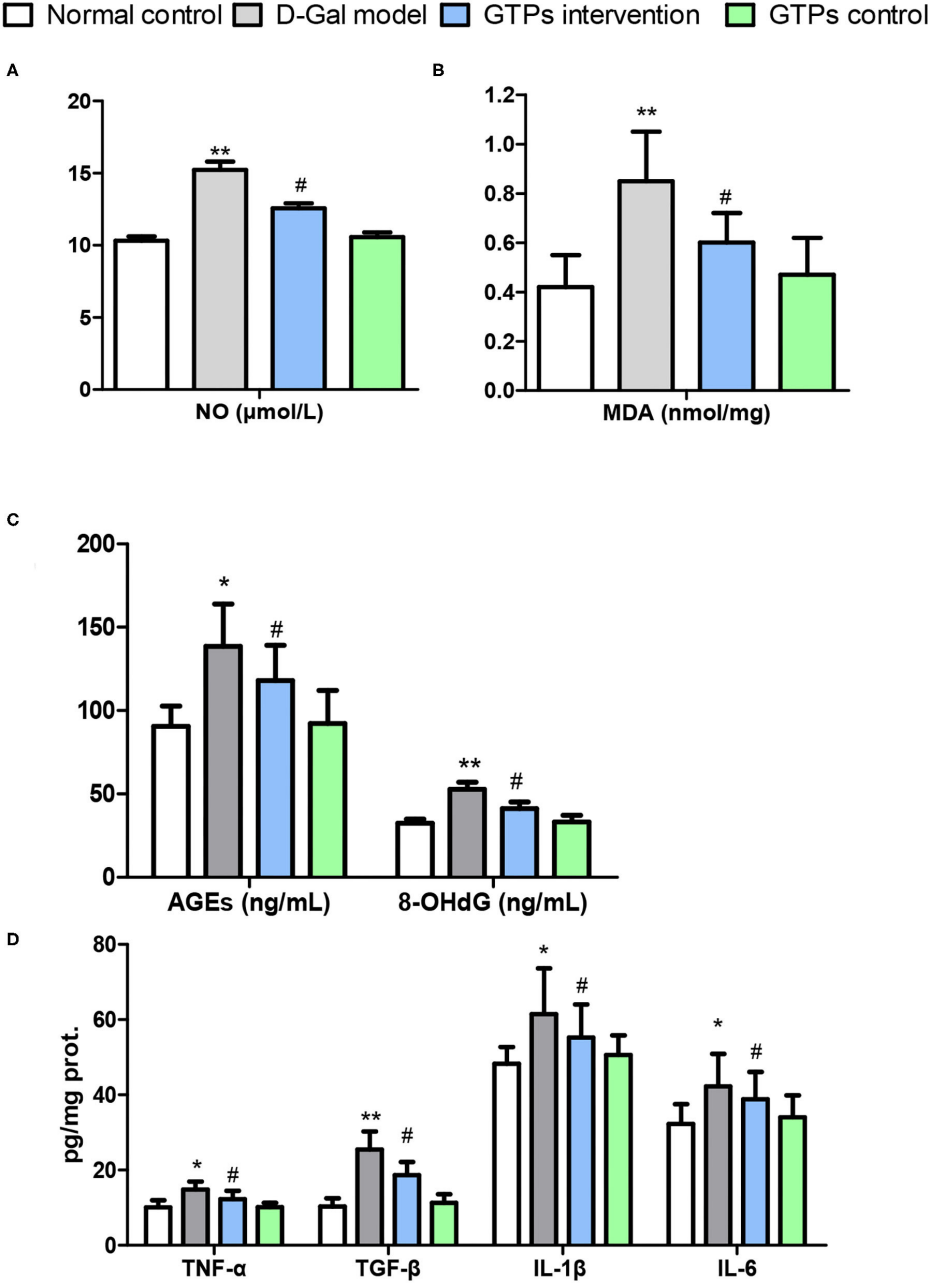
Effects of GTPs on liver oxidative stress markers and inflammatory mediators of D-gal-treated mice. **(A)** Plasma level of NO. **(B)** Hepatic contents of MDA. **(C)** Plasma levels of AGEs and 8-OHdG. **(D)** Hepatic contents of TNF-α, TGF-β, IL-1β, and IL-6. ^*^P < 0.05 and ^**^P < 0.001 vs. control group; ^#^*P* < 0.05 and ^##^*P* < 0.01 vs. D-Gal model group.

In this study, liver antioxidant and oxidative stress markers were screened to explore the protective effects of GTPs against D-Gal-induced liver aging. In the D-Gal model group, antioxidant and oxidative stress marker values were significantly different from those of the normal control group. Hepatic antioxidant markers, namely T-SOD, CAT, GST, GSH, and T-AOC, significantly decreased in the D-Gal-treated group (Figures 1D–F). Furthermore, hepatic oxidative stress and senescence markers, namely MDA, NO, 8-OhdG, and AGE, significantly increased in the D-Gal-treated group (Figures 2A–C). Moreover, these antioxidant markers were significantly increased, and the oxidative stress and senescence markers were significantly inhibited in the D-Gal model mice supplemented with GTPs (Figures 1, 2). These results highlight the regulatory role of GTPs in relation to the redox imbalance and oxidative stress in the liver of D-Gal-treated mice. However, the supplementation of GTPs did not affect hepatic markers of antioxidant, oxidative stress, and senescence.

### Effects of GTPs on Liver Inflammatory Mediators

The hepatic level of TNF-α (proinflammatory cytokine) in the D-Gal-treated mice was significantly higher than that in the normal control group (Figure 2D). Furthermore, the hepatic TNF-α level of the GTPs intervention group was significantly lower than that in the D-Gal model group (Figure 2D). Similarly, the level of hepatic TGF-β was significantly higher in the D-Gal model mouse; furthermore, the hepatic TGF-β level was lower in the D-Gal model mice supplemented with GTPs (Figure 2D). Additionally, the administration of GTPs significantly inhibited the production of both IL-1β and IL-6. However, only GTPs did not affect hepatic inflammatory mediators.

### Effects of GTPs on Hepatic Histopathological Alterations

**Figure 3:**
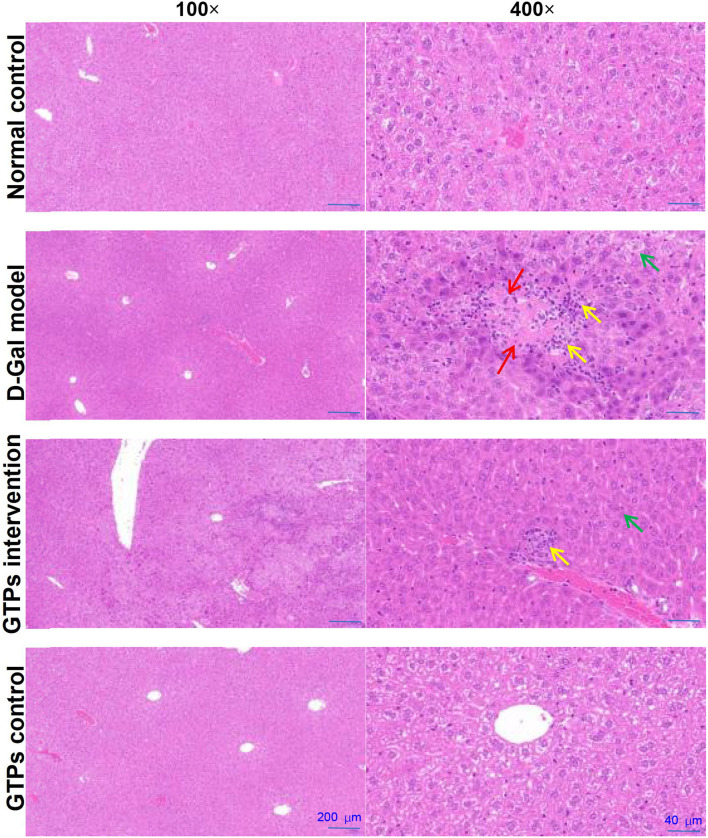
Effects of GTPs on hepatic histopathological alterations in D-Gal-treated mice. Representative HE-stained sections of the liver tissues from rats in each group (100× and 400×). Green arrow indicates edema, vacuoles, and cytoplasmic porosity. Yellow arrow indicates inflammatory cell infiltration. Red arrow indicates degeneration. Scale bars (200 and 40 μm).

The histopathological analysis found that D-Gal caused edema, vacuoles, cytoplasmic porosity, inflammatory cell infiltration, and degeneration in the liver (Figure 3). GTP was able to counteract these harmful effects and reduce the pathological damage of the liver induced by D-Gal (Figure 3). However, only GTPs did not affect hepatic histopathological changes.

### Effects of GTPs on the Hepatic Nrf2 Signaling Pathway

**Figure 4:**
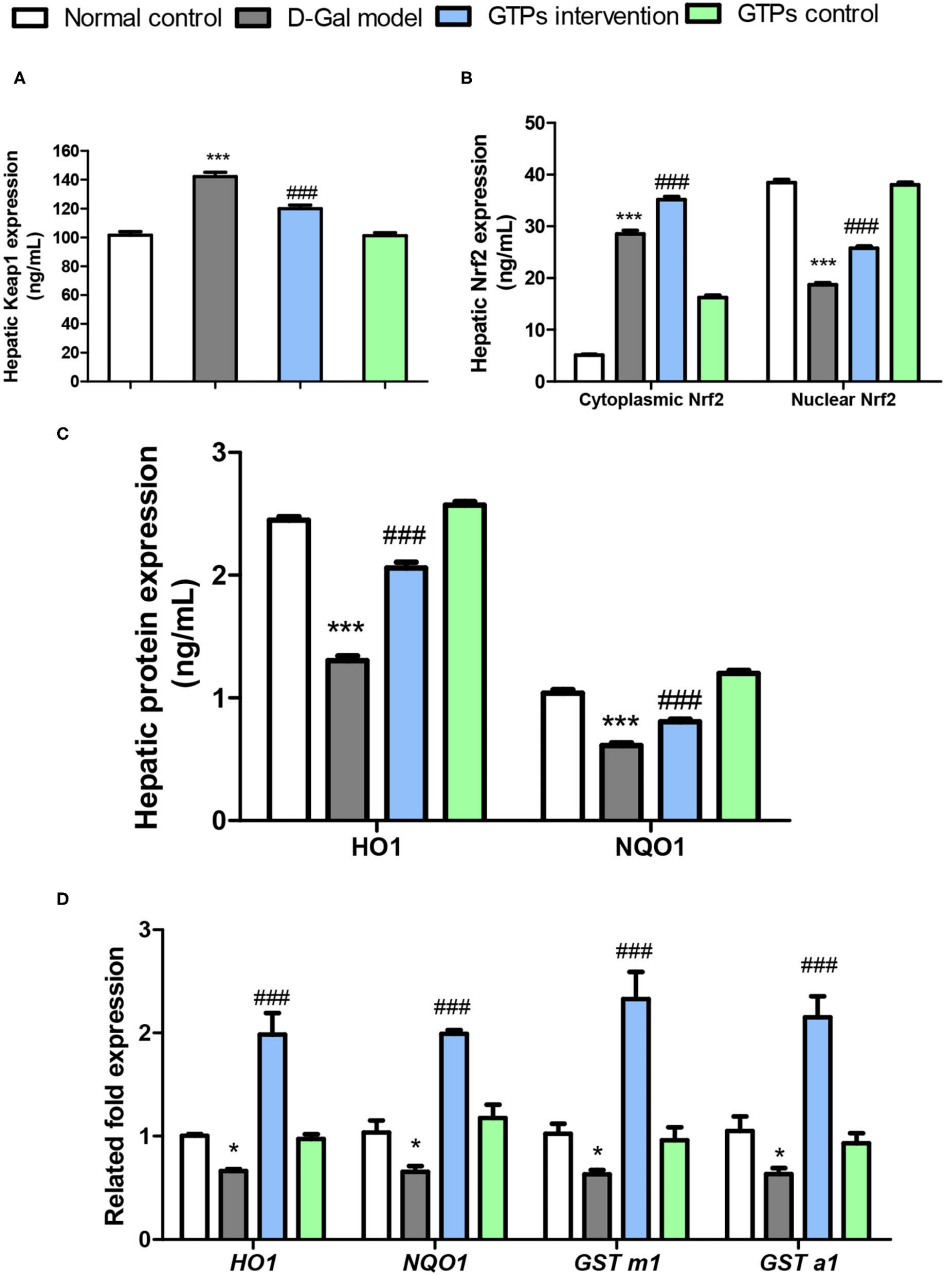
Effects of GTPs on the expressions of Nrf2 pathway in the liver of D-Gal-treated mice. **(A)** Hepatic protein levels of Keap1. **(B)** Hepatic protein levels of cytoplasmic and nuclear Nrf2. **(C)** Hepatic protein levels of HO1 and NQO1. **(D)** Hepatic mRNA expressions of Nrf2-targeted genes. ^***^P < 0.001 vs. control group; ^###^*P* < 0.001 vs. the D-Gal model group.

To investigate the underlying mechanism of the anti-aging effect caused by the administration of GTPs, the signal changes of the Nrf2/Keap1 pathway in mice treated with D-Gal were investigated. Mice treated with D-Gal had significant changes in Keap1 and Nrf2 and its downstream target genes in the liver (Figure 4). Hepatic Keap1 levels were 40% higher in response to D-Gal treatment (Figure 4A). Hepatic Nrf2 (cytoplasmic and nuclear) had a different pattern compared to Keap1 in the GTP-treated group; both cytoplasmic and nuclear Nrf2 levels in the liver were significantly higher in the GTPs-treated group compared within the D-Gal model mice (Figure 4B). Furthermore, the HO-1 and NQO1 protein expression levels in the liver of aged mice were significantly lower (by 47 and 36%, respectively) in the D-Gal model group compared within the normal control group (Figure 4C). The drastic decreases in HO-1 and NQO1 levels were abrogated in the D-Gal model mice supplemented with GTPs (Figure 4C). D-Gal injection significantly downregulated the gene expression of HO-1, NQO1, GST m1, and GST a1 in the liver (Figure 4D). Moreover, the Nrf2-targeted gene expression levels were increased in the D-Gal model mice supplemented with GTPs (Figure 4D).

## Discussion

Aging is a normal physiological phenomenon and is an independent risk factor for various chronic diseases (2). Therefore, discovering and using natural products that may effectively decrease the risk of aging-related diseases are crucial tasks. Oxidative stress and proinflammatory responses are the detrimental causative factors leading to imbalances between oxidative damage and antioxidant function in the development of age-associated conditions (18, 19). Recent evidence suggests that age-related progressive hepatic capacity dysfunction enhances the senescence of hepatocytes (9).

**Figure 5:**
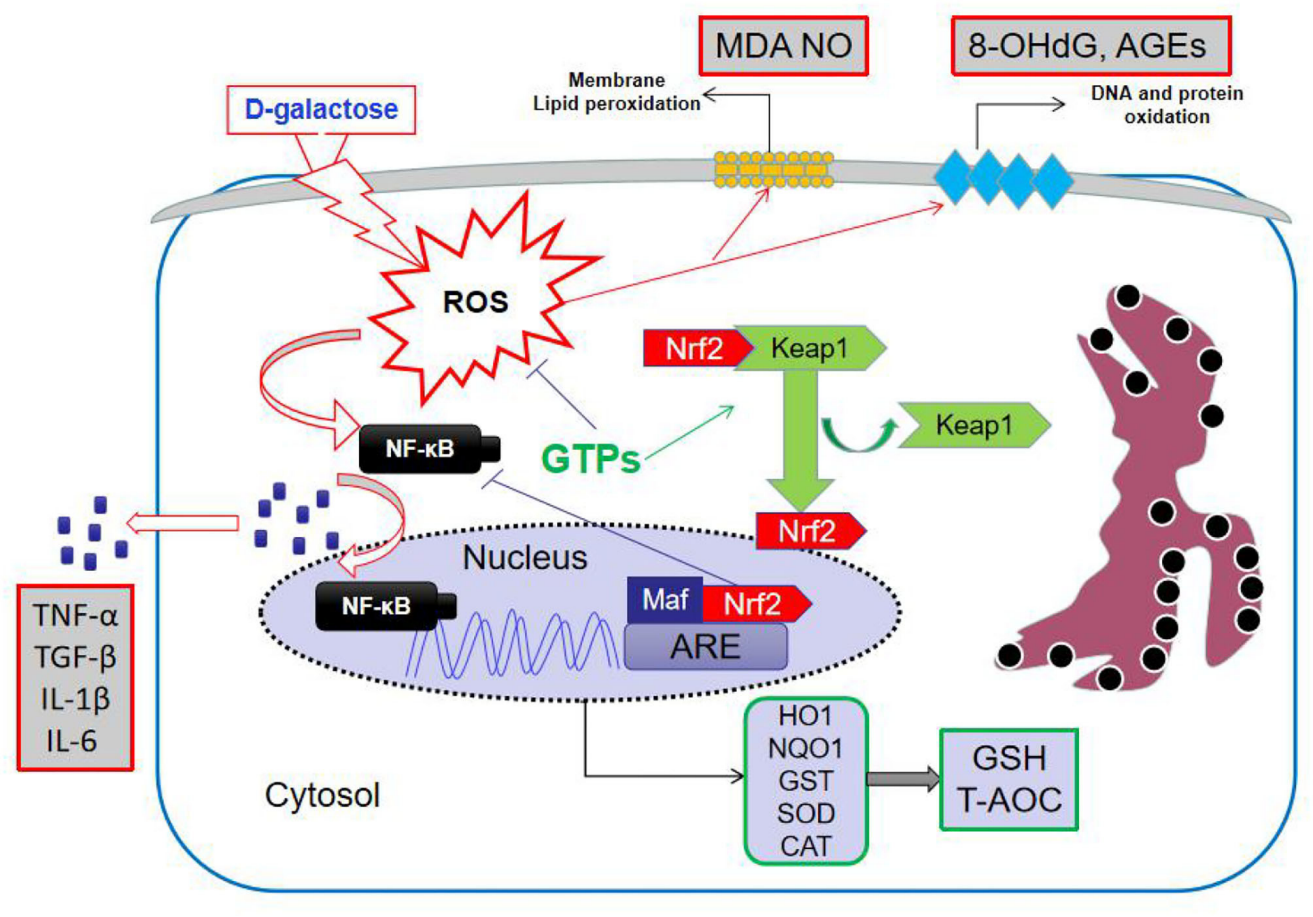
Mechanism of action of GTPs in D-galactose-induced aging in mice liver. GTPs, green tea polyphenols; ROS, reactive oxygen species; MDA, malondialdehyde; NO, nitric oxide; 8-OhdG, 8-hydroxydeoxyguanosine; AGEs, advanced glycation end products; Keap1, Kelch-like ECH-associated protein 1; Nrf2, NF-E2-related factor 2; NF-kB, nuclear factor kappa B; ARE, antioxidant response element; HO-1, hemeoxygenase; NQO1, NAD(P)H:quinone oxidoreductase 1; GST, glutathione S-transferase; SOD, superoxide dismutase; CAT, catalase; GSH, reduced glutathione; T-AOC, total antioxidant capacity; TNF, tumor necrosis factor; TGF, transforming growth factor; IL, interleukin.

D-galactose has been widely applied to induce an aging-like condition in various organs to study different biomarkers (20–22). High doses of D-Gal can induce the formation of hydrogen peroxide, which is invariably linked to ROS, thus leading to hepatic metabolic disorders and, ultimately, liver aging (23, 24). Therefore, in our study, the D-Gal-induced liver aging model in mice was used. The hepatic antioxidant system plays an important role in maintaining the normal liver function, and changes in antioxidants in this system impact the endogenous antioxidant capacity (24). Although the antioxidant properties of GTPs were confirmed using this *in vivo* model, this is the first study to investigate their ability to alleviate the hepatic oxidative stress in D-Gal-treated mice. Thus, our results showed that GTPs can improve hepatic antioxidant capacity and maintain hepatic redox balance during simulated aging. D-Gal significantly increased hepatic damage by changing the levels of plasmatic ALP, ALT, and AST, when compared with the control mice. The increase in aminotransferases in D-Gal-induced liver aging has been reported elsewhere (21). Moreover, plasma TBiL level was remarkably increased in these mice. The catabolism process of bilirubin depends on liver function; therefore, a high level of TBiL reflects hepatocellular dysfunction (25). Consistently, we found that GTPs significantly counteracted liver function abnormalities in D-Gal-treated mice (Figure 1). The proposed mechanism of action of GTPs is shown in Figure 5.

GTPs have exhibited antioxidant effects against oxidative stress and liver damage in different *in vivo* experimental models, such as lipopolysaccharide-induced inflammatory liver injury, azathioprine-induced hepatotoxicity, and carbon tetrachloride–induced hepatotoxicity (26–28). Furthermore, our data corroborate previous observations that exposure to D-Gal can induce hepatic oxidative stress associated primarily with a drastic decrease in the activity of antioxidant enzymes and content of non-enzymatic antioxidants in the liver of aged mice (29). We also found that GTPs effectively decreased oxidative products (MDA and 8-OHdG) in D-Gal-treated mice and increased the activity of endogenous antioxidant enzymes (T-SOD, CAT, GST) and content of antioxidants (T-AOC and GSH) in aging liver tissues, leading to the mitigation of D-Gal-induced impaired liver function. In a previous study with primary cultured rat hepatocytes exposed to 1,4-naphthoquinone, GTPs were not able to counteract the lipid oxidation (i.e., MDA levels) (30).

In an aging pathological state, oxidative stress can induce NF-κβ activation in the liver in addition to evoking direct cellular damage, thereby resulting in the release of proinflammatory cytokines (31, 32). The overexpression and secretion of proinflammatory cytokines negatively affect the hepatocyte's function and further enhance oxidative lesions. Thus, our results showed that the hepatic pro-inflammatory cytokines levels notably increased after D-Gal treatment in mice (Figure 2D), which was in accordance with the histopathological findings (Figure 3). Nonetheless, GTPs not only suppressed the overproduction of these cytokines but also significantly attenuated inflammatory cell infiltration caused by D-Gal in the liver, corroborating the data obtained by Xu et al. (33). Accordingly, the observed protective effect of GTPs on D-Gal-treated mice is likely to have significant beneficial effects on the inflammatory response.

Recent studies have revealed that inflammation-induced AGEs are accumulated in aging tissues of humans and animals, which can serve as a biomarker of organ function (34, 35). Furthermore, previous studies have verified that AGEs might enhance the homeostasis imbalance and age-related clinical diseases *via* increasing the production of ROS and proinflammatory cytokines (36). Hence, it is hypothesized that GTPs could inhibit AGE production in D-Gal-treated mice and thus prevent liver aging. Notably, Nrf2/Keap1 signaling pathway is one of the major intracellular signaling pathways for attenuating oxidative stress–induced liver aging (24, 37). After signal stimulation, Nrf2 was isolated from Keap1, and activated the processing and synthesis of Nrf2 target antioxidative enzymes and non-enzymatic antioxidants, eventually initiating antioxidant response (24, 38, 39). Interestingly, this study found that GTPs increased the expression of Nrf2, HO-1, and NOQ1 proteins in the liver of D-Gal-treated mice. The phenomenon indicated that GTPs were able to mitigate D-Gal-induced liver aging *via* activation of the Nrf2/Keap1 pathway *in vivo*.

In the present study, a D-Gal-induced senescent mice model was used to investigate the protective effects of GTPs on the liver. GTPs upregulated the Nrf2 signaling pathway, maintained a balance in redox and inflammation, reduced cellular oxidative stress and AGE concentration, and improved the activities of antioxidant enzymes in D-Gal-treated mice, thus ameliorating the simulated aging process. Furthermore, histopathological observation revealed that GTPs effectively inhibited D-Gal-induced hepatic pathological changes, highlighting their potential use as a dietary supplement to decrease the effects of liver aging. Further research must be conducted on the potential mechanism by which GTPs activate Nrf2 translocation. Phosphatidylinositol 3-kinase (PI3K)/Akt signaling pathway plays a vital role in the regulation of proliferation, differentiation, and survival (40). Studies have shown that Nrf2 is a target of the PI3K/Akt signaling pathway and the nuclear translocation of Nrf2 requires the activation of the PI3K/Akt signaling pathway (41, 42). Our previous study found that GTPs can activate PI3K/Akt signaling pathway in mice (43); therefore, these results revealed that the PI3K/Akt signaling pathway was involved in Nrf2 activation induced by GTPs. Additionally, whether other redox-associated transcription factors, such as activator protein-1 and NF-κβ, are involved in the regulation of antioxidant enzymes by GTPs requires further studies. Moreover, the structure–activity relationship responsible for the anti-liver aging activity must be identified to explore its moderating effects on the Nrf2/Keap1 pathway.

Other polyphenols have also been reported to reduce D-Gal-induced liver aging through antioxidant and anti-inflammatory mechanisms, such as rambutan peel polyphenols, purple sweet potato polyphenols, ellagic acid, curcumin, and epigallocatechin-3-gallate (20, 44, 45). These results indicate that polyphenols exert beneficial effects on liver aging, and GTPs may be a source of bioactive polyphenols for protecting liver aging due to their rich content and biological activity *in vivo*. Although GTPs have a good protective effect on liver aging, some studies have found that the long-term consumption of tea polyphenol epigallocatechin-3-gallate can cause subacute liver failure in mice, including hepatocyte necrosis along with an abnormal change of blood transaminases, TBiL, and Alb (46). Among the active components of GTPs, anthocyanin, gallic acid, and epigallocatechin-3-gallate have been reported to have potential beneficial effects for the prevention and treatment of age-related liver diseases in mice or rats (47–49). Although we have observed beneficial effects of GTPs in a mouse model of liver aging, it is prudent to state that more studies using *in vitro* (i.e., cell cultures) and *in vivo* (i.e., piglets) protocols are highly required to demonstrate the toxicological safety, dose and time dependency effects, and overall outcomes of the supplementation of GTPs. To date, the consumption of GTPs in powder form or capsules that contain a dose of catechins more than 800 mg/day is not incentivized by international governmental agencies, such as the European Food Safety Authority (EFSA). From a practical standpoint, the dosage in future studies should be pondered: doses higher than those normally consumed in 1-3 teacups a day may cause damage to the outer mitochondrial membrane of hepatocytes (cell injury), and an uncoupling of oxidative phosphorylation may occur, which increases the ROS production and cytokines secretion (50, 51).

In the current study, we found that GTPs exert protective effects against D-Gal-induced liver aging in mice. These results show that GTPs can decrease D-Gal–induced liver dysfunction, histopathological changes, oxidative stress, pro-inflammatory cytokines production, and expression levels of 8-OHdG and AGEs in the liver through regulating Nrf2 signaling pathways. The results highlight the importance of GTP as a natural supplement of anti-aging compounds.

## Data Availability Statement

The original contributions presented in the study are included in the article/Supplementary Material, further inquiries can be directed to the corresponding author/s.

## Ethics Statement

The animal study was reviewed and approved by Animal Care and Ethics Committee at the Jiangsu University of Science and Technology (ethical approval code: 20200302), where the *in vivo* protocol was conducted.

## Author Contributions

DW and DG contributed to the conceptualization and writing the original draft. TW contributed to data curation. DW was responsible for the investigation and resources. ZL was responsible for the methodology. YG visualized and supervised. ZL and DG reviewed and edited the manuscript. All authors have read and agreed to the published version of the manuscript.

## Funding

This research was supported by the Natural Science Foundation of Jiangsu Province (BK20210881) and a talent fund from the Jiangsu University of Science and Technology.

## Conflict of Interest

The authors declare that the research was conducted in the absence of any commercial or financial relationships that could be construed as a potential conflict of interest.

## Publisher's Note

All claims expressed in this article are solely those of the authors and do not necessarily represent those of their affiliated organizations, or those of the publisher, the editors and the reviewers. Any product that may be evaluated in this article, or claim that may be made by its manufacturer, is not guaranteed or endorsed by the publisher.
